# Uncovering employment outcomes for autistic university graduates in the United Kingdom: An analysis of population data

**DOI:** 10.1177/13623613231182756

**Published:** 2023-06-23

**Authors:** Jonathan Vincent, Kevin Ralston

**Affiliations:** 1 Lancaster University, UK; 2 The University of Edinburgh, UK

**Keywords:** autism, college, employment, outcomes, university

## Abstract

International research suggests that increasing numbers of autistic people are entering higher education. Currently, very little is known about this population. For example, the rates of autistic people enrolling at UK-based higher education institutions, the demographics of this population, the subjects they study and particularly their graduate outcomes are unknown. This study is an exploratory analysis of autistic graduate outcomes. We compare outcomes between autistic students, other disabled students and non-disabled students, by sex. The article draws upon population data collected by the Higher Education Statistical Agency in the United Kingdom (*N* = 1,326,416) across the years 2012–2018. Our findings indicate that the academic programmes studied at university by autistic students are more diverse than typically assumed. We also found that graduates make the transition into a range of employment sectors following graduation, but experience persistent and disparities in economic activity and income. We argue that higher education institutions must focus greater attention on developing more robust and effective employment transition support for autistic students and graduates.

**Lay abstract:**

International research suggests that more autistic people are entering higher education. Currently, very little is known about this group in the United Kingdom, for example, we have little information about how many autistic people enrol at UK-based higher education institutions, their backgrounds, the academic programmes they study and what they do once they have graduated. Our study tries to explore these issues by comparing outcomes between autistic students, other disabled students and non-disabled students. We use population data collected by the Higher Education Statistical Agency in the United Kingdom, which included 1,326,416 graduates across the years 2012–2018. Our findings indicate that the degree subjects studied at university by autistic students are more diverse than often people think. We also found that graduates go on to work in a range of employment sectors following graduation but often experience worse outcomes in terms of access to full-time work and worse pay. We argue that universities and colleges must focus greater attention on developing better employment transition support for autistic students and graduates.

## Introduction

Autism is a lifelong neurodevelopmental condition characterised by qualitative differences in social interaction, communication and repetitive behaviours (American Psychiatric Association (APA), 2013). While autism is defined by a core set of traits, there is great phenotypical heterogeneity and diversity in its presentation (Crespi, 2021). Evidence suggests that approximately 50% of autistic individuals have average or above average intelligence (Anderson et al., 2017) and many possess skills including attention to detail, high levels of focus and identifying creative solutions to tasks (Scott et al., 2019). Such traits present opportunities for success in higher education, however, very little is known about autistic students who attend university or college, particularly in terms of background characteristics, academic programmes or employment outcomes including job type or salary. By drawing on population data over 6 years, this study seeks to shed new light on the UK autistic university population to provide baseline evidence that can inform policy and practices in higher education.

### The autistic population in higher education

International evidence indicates that increasing numbers of autistic people are entering university (Bakker et al., 2019, 2022; Jackson et al., 2018), although to date there is little population data to verify exact numbers. White et al. (2011) argue that between 0.7% and 1.9% of American students could meet the criteria for an autism diagnosis and Wei et al. (2017) estimate this to be approximately 16,000 autistic students each year. More recently, Fernandes et al. (2021) reported on The Freshman Survey, a long running survey including the background characteristics of incoming college students at over 1900 institutions in the United States. They estimate that there were 37,026 (0.6%) first-time, full-time autistic students attending colleges and universities in the fall of 2012, 2014, 2016 and 2018. In the United Kingdom, data from the Higher Education Statistical Agency (HESA, 2022) indicates that 16,685 autistic students disclosed their condition to their university in 2020/2021. However, it is likely that the total autistic university population is much larger still.

One of the reasons for this is that many autistic students disclose their autism pragmatically and sometimes not at all (Cox et al., 2017; Sturm & Kasari, 2019). Others, particularly women, will either remain undiagnosed (Estrin et al., 2021), are awaiting diagnostic assessment (Wilson et al., 2016) or experience ‘diagnostic overshadowing’ where pre-existing health conditions, including anxiety, depression or eating disorders, are misapplied to autistic traits, which can delay or prevent diagnosis altogether (McFayden et al., 2023). Moreover, given the high rates of co-occurrence between autism and other neurocognitive conditions such as dyslexia, dyspraxia and mental health conditions (Hossain et al., 2020; Sturm & Kasari, 2019), students’ autism diagnoses are not always specifically captured in university records. All of this leads to a situation where there are limited data on the autistic university population and a high level of underreporting of the condition.

There are only a small number of large-scale studies. These shed some light on the population, including some of the background characteristics. Fernandes et al. (2021) report that autistic students are significantly more likely to be male (69.5%) in contrast to non-autistic students (45.1%). This aligns with the study by Bakker et al. (2022) who present data on prevalence and background characteristics of students’, progression, dropout and degree completion from a public university in the Netherlands. Their analysis compared autistic (*n* = 101), students with other conditions such as attention deficit disorder / attention deficit hyperactivity disorder and dyslexia (*n* = 2465), and students with no declared disability (*n* = 25,077). They reported 71.7% of the sample of autistic students as male and that autistic students were also typically older compared to non-autistic peers (Bakker et al., 2022). Sturm and Kasari (2019) report that a large proportion of autistic students came from high-income families (> 100,000; 42%); a trend that likely represents the increased access that more affluent families have to diagnostic services (Skylark & Baron-Cohen, 2017). While this limited literature offers important insights, much more data are required to better understand the diverse autistic population, including non-binary students, those with co-occurring conditions, or women who have learned to camouflage their autistic traits (Estrin et al., 2021).

Potential stereotypes are also present with respect to the academic programmes studied by autistic students. Given that autistic students can present various academic skills and abilities in areas such as mathematics (Dillenburger et al., 2013; Krzeminska & Hawse, 2020), there is an assumption that they are naturally drawn to Science, Technology, Engineering or Mathematics (STEM) programmes. Indeed, there is some evidence for this (see the studies by Anderson et al., 2017;) including the National Longitudinal Transition Study-2 (NLTS-2), which reports that autistic students have the highest STEM participation rates among 11 disability categories at 34% (Wei et al., 2014). Even higher rates are reported in the study by Bakker et al. (2019), which indicates that 55.7% autistic students are enrolled in STEM programmes. However, such figures only represent those students who have disclosed a diagnosis and so it is likely that there are many more autistic students who remain undiagnosed and study non-STEM subjects. Gaining an accurate picture of the range of academic programmes studied by autistic students in the United Kingdom could encourage institutions to consider their accessibility.

Finally, there is limited but conflicting evidence with respect to outcomes for autistic students and graduates. One of the few studies, such as by Bakker et al. (2022), compare progression and completion of Bachelor’s degrees across 53 programmes and report that credits, grade-point average, resits, degree completion, and drop-out rates were largely the same when comparing autistic and non-autistic groups. This is positive but in contrast to data from the NLTS-2 analyses, which indicate that American autistic students are less likely than their peers to complete higher education (Wei et al., 2014). It is worth noting, however, that the study by Bakker et al. (2022) examines a single institution in the Netherlands compared to NLTS-2, which includes a much more diverse sample of 2- and 4-year colleges across the United States and so direct comparisons are problematic. In terms of employment outcomes, there is very little data available. The Association of Graduate Careers Advisory Services (AGCAS) (2022) in the United Kingdom reports that of the disabled graduates, autistic graduates have the lowest proportion of full-time employment (57.5%) with only around a third working in roles that reflect their level of qualification. These findings are consistent with the small number of qualitative studies that also relate poor employment outcomes among autistic graduates. These report practical difficulties throughout the recruitment process and attitudinal barriers among hiring managers often leading to unemployment or mal-employment (Vincent, 2020; Vincent & Fabri, 2022). Thus, it appears that where autistic graduates complete their university programmes, they experience barriers in accessing full-time employment in graduate-level roles, however, data are scarce.

Stark et al. (2021) call for prospective, large population-based studies examining outcomes of autistic students. There is also a need to understand this population in context. Lord et al. (2020) argue that analysis of autistic groups requires meaningful comparative context or any negative outcomes may be over-emphasised and autistic students further stigmatised as a result. Our analyses seek to ameliorate these gaps by providing comparative context with large and robust data over the period 2012–2018. This study uses the following guiding research questions:

1. What are the background characteristics of the autistic population in higher education in the United Kingdom?
  (a) How many graduates report an autistic identity?
  (b) What is the makeup of the autistic graduates with respect to reported sex?
  (c) What academic programmes do autistic students complete and how do these compare to other non-autistic disabled graduates and non-disabled peers?
2. What are the employment outcomes for autistic graduates?
  (a) What kinds of job roles do autistic graduates do following university and how do these compare to other non-autistic disabled graduates and non-disabled peers?
  (b) What level of salary do autistic graduates achieve following university compared to other non-autistic disabled graduates and non-disabled peers?
  (c) Do autistic employment outcomes vary by the reported sex of the graduate?

## Methods

### Instruments

The data for this study are provided by the Higher Education Statistics Agency (HESA). The period covered is from the academic year 2012 through to 2018. The data are from two cross-sectional surveys. The first is the Destinations of Leavers from Higher Education survey (DLHE), which covers years 2012/2013 through 2016/2017. This survey was replaced by the Graduate Outcomes Survey (GOS) in 2017/2018. Both surveys gather information on employment outcomes along with socio-demographic data about graduates. This includes information on economic-activity, earnings and whether graduates went on to further study. Other relevant information is also collected, such as information on the undergraduate degree taken. Universities in the United Kingdom are required to collect these data as a condition of their funding.

There are some differences between the DLHE and the GOS that may have implications for analyses. Data regarding employment outcomes in the DLHE were collected from graduates via telephone interviews at a time point 6 months following completion of undergraduate studies with a targeted response rate of 78% (HESA, 2018). The equivalent data from the GOS were collected 15 months after the completion of studies. The GOS is an online survey where graduates are contacted via email and reports a response rate of around 50%. This may account for a difference in sample size of the GOS in comparison to the DLHE. It is possible that the difference in timing of when post-graduation information is collected between the DLHE and GRO could be associated to substantive outcomes around employment and further study. If this were the case, it could lead to false comparisons being made.

Although these are different data collection tools, the information gathered across the DHLE and GOS is similar or identical. Proportions by response category on the variables analysed are very similar between the surveys. This is the situation for key variables such as disability status, sex and programme of study. In checking the data there were no marked or obvious patterns of disparities in responses between the two surveys by categories of analysis (see Supplementary Tables). Responses to the GOS fit patterns of response and trends evident in the DLHE surveys (e.g. an incremental increase in the proportion self-reporting as autistic). Therefore, although the survey instruments differ in mode and timing, the information gathered is comparable and the data collected appear to be robust between the instruments.

The period of analysis begins with 2012/2013 survey. This was the point at which HESA began collecting data on graduates with ‘social communication difficulties and autism spectrum conditions’. The last available year of data collected prior to the COVID-19 pandemic is 2017/2018. The global response to COVID-19 has been recognised as substantially impacting graduate outcomes (Treviño-Reyna et al., 2021). This has been highlighted as particularly impactful for graduates from disability, minority and protected characteristic groups (Tomlinson et al., 2021). The COVID-19 affected period therefore requires separate and special consideration and is not included in this study.

### Ethics

Ethical approval for use of the secondary data set was obtained from the first author’s institution [RECEDU00058]. While our secondary analysis responds to the research priorities of the autistic community with respect to developing knowledge about trends in employment (Nicholas et al., 2017; Pellicano et al., 2014), at this exploratory stage, we acknowledge a lack of community involvement. However, we would aim to address this in future studies, particularly where these include the collection of primary data.

### Sample and variables

In principle, the surveys include all UK university graduates across the period from 2012 to 2018. The total number who self-reported their postgraduate outcomes across the time period was 1,326,416. Of those, 1,140,637 (slightly less than 86%) reported having No Disability; 180,133 (13.6%) identified as having an ‘Other Disability’; and 5646 (0.4%) reported social communication/autistic spectrum disorder. Graduates self-identified their disability status. No formal diagnostic information is available and there is no obligation on a respondent to disclose a disability. As with other studies (e.g. Bakker et al., 2019; Fernandes et al., 2021; Sturm & Kasari, 2019), the proportion identifying as autistic is small. In our study, this proportion increased over time from around 0.5% (2012/2013) to around 1% (2017/2018). Approximately four times more male graduates (0.8%) identify as autistic compared to female respondents (0.2%), which is again resonant with other evidence. The number of female graduates who report being in the Other Disability category also rose across the period.

In addition to disability status, the analysis focuses on several key outcomes. These are examined by the reported sex of the graduate. While evidence suggests that transgender and non-binary identities are particularly common among the autistic community (Stagg & Vincent, 2019), the survey question requests the biological sex (rather than gender identity) of the respondent and is coded male or female. Academic programme of study is included in our analysis. This is based on the Joint Academic Coding System (JACS 3.0). This groups 164 principal subjects into 19 subject areas: for example, biological sciences, creative arts and design or education (see Table 1). Income and earnings information is available in a ‘salary’ variable. This is the graduate’s annual pay to the nearest 1000 British pounds, before tax. For these analyses, the classification is organised into categories of £20,000 or less, £20,001–£25,000, and a £25,001 and above category (see Table 3). For context, the Office for National Statistics (2023) estimate median household disposable income in the United Kingdom to be £32,300 with the median income for graduates estimated to be £30,921 and £29,000 in 2014 (Institute for Student Employers, 2022). An employment sector variable is based on the UK Standard Industrial Classification of Economic Activities 2007 (Prosser, 2009). There are 16 industrial sectors, which include, for example, construction, manufacturing and education (see Supplementary Tables 7–12).

**Table 1.** Degree subject area by self-identified autism status of those working full-time or part-time.

	Men				Women			
Degree subject area	Autism	Other disability	No known disability	Total	Autism	Other disability	No known disability	Total
Medicine & dentistry	10	1383	10,374	11,767	7	2032	13,878	15,917
	0.2%	2%	2%	2%	0.6%	2%	2%	2%
Subjects allied to medicine	77	4158	26,472	30,707	82	14,103	80,463	94,648
	2%	6%	5%	5%	6%	13%	13%	13%
Biological sciences	316	6358	43,406	50,080	129	12,178	68,653	80,960
	7%	9%	9%	9%	10%	11%	11%	11%
Veterinary science	1	101	530	632	2	322	1330	1654
	0.02%	0.1%	0.1%	0.1%	0.2%	0.3%	0.2%	0.2%
Agriculture & related subjects	38	857	3844	4739	27	1471	7049	8547
	0.9%	1%	0.8%	0.8%	2%	1%	1%	1%
Physical sciences	336	4050	27,439	31,825	51	3590	20,875	24,516
	8%	6%	6%	6%	4%	3%	3%	3%
Mathematical sciences	204	1410	13,336	14,950	35	1163	8984	10,182
	5%	2%	3%	3%	3%	1%	1%	1%
Computer science	689	4993	36,505	42,187	45	1314	8535	9894
	16%	7%	7%	7%	4%	1%	1%	1%
Engineering & technology	270	6361	57,829	64,460	31	1635	11,415	13,081
	6%	9%	12%	11%	2%	2%	2%	2%
Architecture, building & planning	50	2851	19,991	22,892	5	1508	10,221	11,734
	1%	4%	4%	4%	0.4%	1%	2%	2%
Social studies	294	6801	45,078	52,173	106	13,028	70,582	83,716
	7%	10%	9%	9%	8%	12%	11%	11%
Law	79	2107	16,221	18,407	28	3485	25,793	29,306
	2%	3%	3%	3%	2%	3%	4%	4%
Business & administrative studies	272	7793	77,300	85,365	57	8215	77,425	85,697
	6%	11%	15%	15%	5%	8%	12%	11%
Mass communications & documentation	256	2254	14,256	16,766	61	3068	19,321	22,450
	6%	3%	3%	3%	5%	3%	3%	3%
Languages	231	2429	17,907	20,567	111	6632	42,928	49,671
	5%	3%	4%	4%	9%	6%	7%	7%
Historical & philosophical studies	323	4135	23,239	27,697	84	5393	26,873	32,350
	7%	6%	5%	5%	7%	5%	4%	4%
Creative arts & design	814	9040	36,749	46,603	288	16,194	59,896	76,378
	19%	13%	7%	8%	23%	15%	9%	10%
Education	92	3656	29,588	33,336	112	12,642	79,089	91,843
	2%	5%	6%	6%	9%	12%	12%	12%
Combined	14	394	2699	3107	7	884	4280	5171
	0.3%	0.6%	0.5%	0.5%	0.6%	0.8%	0.7%	0.7%
Total	4366	71,131	502,763	578,260	1268	108,857	637,590	747,715
	100%	100%	100%	100%	100%	100%	100%	100%

Source: Destinations of Leavers from Higher Education survey (DLHE) 2012 to 2017 and the Graduate Outcomes Survey (GOS) in 2018.

Pooled HESA data 2012–2018, First row has frequencies and second row has column percentages.

An activity variable indicates the most important activity as defined by the respondent. This is similar to a standard economic activity variable used by the Labour Force Survey (Office for National Statistics, 2022). In our analyses this comprises of full-time and part-time employment categories, an employed and/or studying category, an unemployment category and a general ‘other’ category (see Table 2). Full-time and part-time work includes those who indicated their most important activity was working, but who are not also involved in study or research. The employment and/or study category includes those who indicated any combination of employment with study, or study alone as their main activity. It contains those who indicated their most important activity was full-time or part-time study. It also includes respondents who say they are in work but where they indicated they were also studying (e.g. primarily in work but also studying). Unemployed includes those who indicated that their most important activity was unemployed or who were currently unemployed but due to start work or study. HESA indicates that the ‘Other’ category includes those whose most important activity was either taking time out to travel, or doing something else, for example taking on a caring responsibility.

**Table 2.** Economic activity of graduates 2012 to 2018 by autism for men and women.

	Ft-work	Pt-work	Work + study	Unemployed	Other	Total
			Men			
Autism	1514	748	1070	732	302	4366
	35%	17%	25%	17%	7%	100%
Other disability	41,374	9436	11,635	4893	3793	71,131
	58%	13%	16%	7%	5%	100%
No known disability	353,027	59,091	59,410	15,675	15,560	502,763
	70%	12%	12%	3%	3%	100%
Total	395,915	69,275	72,115	21,300	19,655	578,260
	68%	12%	12%	4%	3%	100%
			Women			
Autism	409	264	346	140	109	1268
	32%	21%	27%	11%	9%	100%
Other disability	61,206	18,186	17,578	5418	6469	108,857
	56%	17%	16%	5%	6%	100%
No known disability	417,763	100,804	79,623	16,150	23,250	637,590
	66%	16%	12%	3%	4%	100%
Total	479,378	119,254	97,547	21,708	29,828	747,715
	64%	16%	13%	3%	4%	100%

Source: Destinations of Leavers from Higher Education survey (DLHE) 2012 to 2017 and the Graduate Outcomes Survey (GOS) in 2018.

First row has frequencies and second row has row percentages.

### Analysis

The data in this study were analysed descriptively. This approach is necessary to provide a baseline understanding of the degree subject choice and outcomes of autistic graduates in relation to their non-autistic peers. Our descriptive analytic approach follows other labour market studies; for example, MacMillan (2014) who examined intergenerational unemployment in the United Kingdom and Jones (2021), who similarly quantifies the level of labour market disadvantage experienced by disabled people.

Data management and analysis was undertaken using Stata^®^ 17.0. The analyses are presented by pooling the data for the full 6-year period, 2012–2018. Disaggregated responses to the key outcome variables, from the graduates self-reporting as autistic, are reported for each survey year in the Supplementary Materials. This highlights basic consistency in proportions by response category for each outcome by survey year, indicating that pooling the data does not generally hide underlying temporal trends. An interesting exception to this is unemployment, noted in the results section below. Although there is a relatively large number of individuals self-reporting as autistic, disaggregation of the data by categories leads to sparseness and empty cells. For example, when examining occupational sector, only a proportion of the autistic graduates are in employment (*n* = 2935). Of this, a smaller proportion are female (*n* = 673). Disaggregated further, across the 21 occupational sectors and by year, would lead to excessive sparseness.

The relationship between self-reported disability and a set of key outcome variables by sex is considered in the analyses. Comparisons are drawn between respondents who reported No Disability, those who identified as having an Other Disability, and those who identified as autistic. This begins with a breakdown of the undergraduate programme of study (degree subject area) undertaken by autistic graduates, compared to the comparison groups. Following this, an examination of the economic activity of the autistic group in comparison to the other groups is then provided. The employment destinations of the graduates are elaborated, outlining the industrial sector where the autistic graduates are most likely to be employed, in comparison to the non-autistic groups. Finally, the level of income of the autistic group in comparison to the other non-autistic groups is elaborated.

## Results

### Academic programme

A number of academic programmes indicate higher proportions of autistic students compared with those reporting Other Disability and No Disability (see Table 1). This is particularly the case with respect to creative arts and design, where autistic females are more likely than the males to take these degrees (19% of autistic males and 23% of autistic females). Autistic representation in these subjects is almost three times higher as a proportion than students with No Disability (7% for males and 9% for females). Autistic students are similarly over-represented in computer science. Around 16% of autistic males and 4% autistic females had graduated with a degree in this academic programme compared to 7% of males and 1% of females with No Disability, respectively.

By contrast, there are academic programmes with lower proportions of autistic students. Interestingly, these include STEM subjects such as Engineering and Technology (6% autistic males compared to 12% males with No Disability) and Allied Medicine degrees where the percentage of autistic female graduates was less than half (6%) as compared to females with Other Disability (13%) and females with No Disability (13%). Veterinary science followed by medicine and dentistry were the academic programmes that were least studied by autistic graduates (see Supplementary Tables 1 and 2). Many had similar proportions of autistic and non-autistic graduates; this included, mathematical and physical sciences, law and language degrees (see Table 2). Overall, there are substantial differences evident in the degrees taken by autistic students in comparison to the Other Disability and No Disability groups.

### Economic activity

Table 2 denotes the main activity of respondents and compares autistic graduates with Other Disability and No Disability groups. There is a clear pattern identifying autistic graduates in less advantaged activity categories. For example, 68% of those with No Disability were in full-time employment, but only 34% of those who identified as autistic recorded this outcome. This is also substantially lower than those who report an Other Disability and working full time (57%). Autistic graduates were over twice as likely to report being unemployed (15%) compared to graduates with an Other Disability (6%). While they are five times more likely to report being unemployed than their peers with No Disability (3%). This, however, declined over time, where levels of unemployment for autistic respondents fell from 20% in 2012/2013 to 12% 2017/2018 (see Supplementary Tables 3 and 4). Study or study-combined-with-work was the second most frequent activity indicated by autistic respondents (25%). This was more than twice as high proportion in the No Disability group who were studying or studying while working (12%).

There are sex-based trends with respect to economic activity (see Table 2). Outcomes appear slightly less favourable for males with autism. For example, 17% of autistic males reported being unemployed compared to only 7% of those with Other Disabilities and 3% of males with No Disabilities. By contrast, only 11% of autistic females were unemployed. This was still more than twice the proportion of those with Other Disabilities (5%) and more than four times that of females with No Disabilities (2.5%). There was less of a contrast by sex for those in full-time work. Of autistic males, 35% were in full-time and 32% of autistic females were in full-time work. Again, there are considerable differences in the main activity that graduates with autism are doing following their studies, in comparison to their non-autistic peers.

#### Employment destinations

Autistic graduates are employed in all sectors of the economy (see Supplementary Tables 7 and 8). The education sector is the most frequent destination for autistic graduates (18%). ‘Education’ denotes a range of occupational groupings, including those working in schools, higher education or informal educational settings. The proportion of autistic graduates entering the sector is broadly equivalent to the Other Disability (19%) and No Disability (19%) groups. More female autistic graduates worked in education (26%) compared to autistic males (15%). The proportion of autistic females working in education was also higher than the proportion of those from the Other Disability (23%) and No Disability (22%) groups.

Across the 6-year period of reporting, 15% of autistic graduates reported employment in wholesale and retail, with proportions by sex at similar levels (14% male and 15% female). This is, however, noticeably higher than the proportions from Other Disability (10%) and No Disability (10%) groups who were working in the sector. In 2017/2018, the percentage of autistic graduates in retail fell to 11%, perhaps denoting declining employment opportunity in the sector.

There were also unanticipated patterns of employment by sector. Counter to stereotypes around autistic people preferring to engage in activities involving numeracy or computer programming, rates of autistic graduates entering the professional, scientific or technical sector roles (broadly STEM occupations) across the period of observation was only 8%. This was lower than both Other Disability (11%) and No Disability (12%) groups. Almost twice as many (9%) male autistic graduates entered this sector compared to female autistic graduates (5%).

It is also notable that 13% of autistic graduates entered occupational roles in the information and communication sector in comparison to the Other Disability (7%) and No Disability (7%) groups. This seems counter to expectations, given difficulties with social communication are core criteria for an autism diagnosis. Finally, nearly twice as many autistic graduates by proportion (9%) entered the arts and entertainment sector compared to 6% graduates in the Other Disability and 4% in the No Disability groups. Overall, the evidence shows that employed autistic graduates are distributed in a diverse range of different sectors of the economy.

#### Income

Table 3 shows the reported income (salary) of graduates working full-time for the pooled 2012–2018 period. This shows autistic graduates were less likely to earn over £25,001 and more likely to earn below £20,000 compared to graduates with Other Disabilities and those with No Disabilities. There is, however, a proportion of information missing. The economic activity variable indicates that *n* = 2937 of autistic graduates are in either full-time or part-time employment, with an additional number studying and employed. Income information is only available for *n* = 1095 autistic individuals in full-time employment and so conclusions must be drawn more tentatively. This may represent reluctance to report income.

**Table 3.** Income of those working full-time, by whether an individual self-identified with autism by men and women 2012 to 2018.

	Men				Women			
	< £20,000	£20,001–£25,000	> £25,001	Total	< £20,000	£20,001–£25,000	> £25,001	Total
Autism	397	227	239	863	99	67	66	232
	46%	26%	28%	100%	43%	29%	28%	100%
Other disability	7841	6471	8536	22,848	14,355	11,559	9736	35,650
	34%	28%	37%	100%	40%	32%	27%	100%
No known disability	59,991	49,162	82,197	191,350	94,078	66,178	73,228	233,484
	31%	26%	43%	100%	40%	28%	31%	100%
Total	68,229	55,860	90,972	215,061	108,532	77,804	83,030	269,366
	32%	26%	42%	100%	40%	29%	31%	100%

Source: Destinations of Leavers from Higher Education survey (DLHE) 2012 to 2017 and the Graduate Outcomes Survey (GOS) in 2018.

First row has frequencies and second row has row percentages.

Of the available income data, differences are starker for female graduates compared to male graduates overall. Male graduates were more likely to report having a job earning more than £25,001 (42%) compared to female graduates (31%). However, at 46%, male autistic graduates were the most likely of any group to earn £20,000 or less. Of male autistic graduates, 28% earned £25,001 or above compared to 37% of those with No Disabilities (9% difference). The equivalent difference for female graduates is only 3%. Males with No Disabilities were the most likely to earn more than £25,000 (43%). In sum, well-established patterns of sex-based differences in earnings are apparent among graduates in full-time employment with autistic graduates experiencing additional disadvantaged in comparison to their non-autistic peers.

## Discussion

By drawing on large-scale population data and contextualising these in relation to non-disabled graduates and those with other disabilities (Lord et al., 2020; Stark et al., 2021), this study sheds new light on the UK autistic university population, including background characteristics, academic programmes studied, occupational destinations and economic activity outcomes. We found that the percentage of graduates self-reporting to be autistic was 0.43% of the total sample, although this was nearer to 1% by 2017/2018, which is around the prevalence rate (1.1%) reported for the adult population in the United Kingdom (Brugha et al., 2012). This finding is similar to the estimates by White et al. (2011) of between 0.7% and 1.9% of college students and corresponds with other university-based studies (Bakker et al., 2019; Fernandes et al., 2021; Sturm & Kasari, 2019). However, we suggest that the ‘true’ population is likely to be larger still. One reason is that the mode of data collection in the DHLE survey was telephone interview. This is a form of communication that many autistic people find difficult and may be likely to avoid (Howard & Sedgewick, 2021). It may also be the case that autistic graduates are less likely to self-report the diagnosis in a labour market context where they are looking for work (Romualdez et al., 2021). Four times more male graduates reported to be autistic compared to females, which again reflects the UK national prevalence rates (male 2% / female 0.3%) by Brugha et al. (2012) and is in line with other higher education studies (Bakker et al., 2019; Fernandes et al., 2021; Sturm & Kasari, 2019). However, as others suggest, the disparity might be due to poorer access to diagnostic services, misdiagnosis and evidence of a higher threshold for autism diagnosis among females (Mandic-Maravic et al., 2015; Young et al., 2018). Moreover, our study did not capture data for those who are gender non-conforming (Stagg & Vincent, 2019). So, while these findings form a useful baseline, there continues to be a need for more accurate evidence about the numbers and background characteristics of autistic students in UK universities.

Our data did show the varied range of academic programmes that autistic graduates completed. Unlike other studies, which indicate strong representation on STEM programmes in the United States (Anderson et al., 2017; Shattuck et al., 2014; Wei, et al., 2014) and the Netherlands (Bakker et al., 2019), our UK findings reported low levels of those who graduated from other STEM subjects such as Engineering and Technology, Medicine, Dentistry, Allied Medicine, or Veterinary Science although proportionately high levels of participation in Computer Science. The small numbers studying medicine echo the findings from the study by Turner et al. (2021) who find that medical students in the United Kingdom have significantly lower levels of autistic traits compared to other STEM students and non-STEM students and might, therefore, be less drawn to person-oriented academic programmes or careers. While this could be the case, differential rates could also be due to under-reporting, where autistic graduates in these particular fields did not participate in the data collection. What our data do show is great diversity among the kinds of programmes studied by autistic people at the university. Illustrative of this is the finding that almost three times as many autistic graduates reported completing Creative Arts and Design programmes compared to those with No Disability. Such evidence is useful for challenging the STEM stereotype often applied to autistic people (Gaeke-Franz, 2022), which undermines the heterogeneity of the population and downplays the potential for creativity.

Regarding employment destinations, we found that the education sector was the most frequently reported by autistic graduates across the 6 years. More than a quarter of all female autistic graduates worked in education, which can include early years’ settings, public schools, university or informal settings. This trend resonates with the study by Wood and Happé (2021) where of *n* = 149 autistic teachers or education staff surveyed, 80% identified as female. It also echoes the study by Cheriyan et al. (2021) who report that autistic students were more motivated to seek a role in higher education than non-autistic students. They also found that female autistic students tended to opt for jobs, which helped others compared to their male autistic peers. This runs counter to the claim by Turner et al. (2021) that autistic students might be less likely to pursue person-focused career paths and could indicate that education offers a professional environment where autistic graduates feel able to utilise knowledge and skills acquired at university. In fact, StEvens (2022, p. 2) suggests that their ‘autistic experience gives an advantage in implementing inclusive education practices’.

We found that rates of autistic graduates entering STEM job roles were also lower than both Other Disability and No Disability across the 6 years, which follows the trend identified in the academic programmes studied. This diverges from the wider literature that highlights the ‘natural alignment between AS strengths and STEM industry needs’ (Krzeminska & Hawse, 2020, p. 241) exemplified by various recruitment partnerships with technology companies such as Google, Microsoft, SAP, Nokia, CISCO, IBM, among others (Hayward et al., 2019). The lower rates of STEM employment in our study might, again, indicate under-reporting or that, despite widespread recruitment in this sector, it is not as inclusive as others for autistic graduates. It could also be that STEM-based occupations require different skills, or further experience, that autistic graduates struggle to acquire or articulate at interview. Future studies might seek to uncover the rationale that autistic graduates provide for the career choices they make and the potential barriers that they face in accessing particular sectors.

Importantly, our data demonstrate that economic activity outcomes are consistently poorer for autistic graduates over the 6 years. Half as many autistic graduates were in full-time employment compared to their non-disabled peers, which concurs with a recent UK report from the Association of Graduate Careers and Advisory Services (AGCAS, 2022) showing that autistic graduates were the least likely to be in full-time employment of any disabled group in higher education. Other studies among the adult autistic population also report over-representation of part-time working (Harvery et al., 2021; Ohl et al., 2017) and especially among female autistic adults (Hayward et al., 2018). Part-time employment can be disadvantageous for autistic adults, as it is often comparatively low-skilled and low-paid, and potentially limits opportunities for career advancement (Müller et al., 2003). However, working part-time might also be a preference for autistic graduates used to generate greater flexibility for other interests or to manage burnout from full-time work perceived as ‘stressful and exhausting physically and mentally’ (Hayward et al., 2019, p. 301).

While Bakker et al. (2022) report that autistic graduates often leave university with comparable qualifications to their non-disabled peers and those with other conditions, our analyses demonstrate how these credentials are comparatively less likely to be converted into positive employment outcomes. Consistent with the recent AGCAS (2022) report, we found that autistic graduates are more likely to earn less than their non-autistic peers and well below the median salary for graduates in the United Kingdom at £30,921 in 2022 or £29,000 in 2014 (Institute for Student Employers, 2022). This might be the result of mal-employment, where graduates are over-qualified for the roles (Remington & Pellicano, 2019; Vincent, 2020; Vincent & Fabri, 2022) or gaining work in sectors such as retail, which are more precarious and poorly paid. When sex is taken into account, there exists particular disadvantage among female autistic graduates who are the most likely to earn < £20,000 out of any of the groups. Such findings are a reminder of wider intersectional analyses where the pay of people with more than one disadvantaged identity is lower than that of people with a single disadvantage and that there is greater variability in female pay on the basis of other identities (ethnicity, disability) than there is between females and males (Pettinicchio & Maroto, 2017).

Finally, these data help to make the case for changes to policy and enhanced funding for autistic students in higher education. University personnel should consider how they can maximise the inclusivity of their student offer to support this group across all academic programmes. This might include providing an academic advisor, peer mentoring or making modifications to teaching and assessment procedures (Hillier et al., 2018). Evidence also suggests ensuring that disability services, careers services, and academic faculties are connected is important for developing systematic support systems (Accardo et al., 2019). Our findings identify the specific gap regarding employment outcomes and so institutions might develop programmes that develop the skills required to find and achieve graduate employment. Such training might include enhanced careers guidance throughout university (Pesonen et al., 2021) with a focus on self-advocating strengths and capacities (Hedley et al., 2017; McDowall et al., 2023).

## Limitations

While we report on a large population sample, it is recognised that the data used in this study are self-reported and only reflect the outcomes of graduates in the United Kingdom between the years 2012 and 2018. Moreover, as with any secondary analyses, we were limited by the variables on the original survey. Future studies might extend the measures and the timespan used to capture more granular and up-to-date outcomes. A further limitation of our data set is that for the years 2012–2017 economic activity and earnings were collected approximately 6 months after successful completion of study, whereas in 2017/2018, these data were collected after 15 months. This could not only allow for a longer period of potential career success following graduation, but also the potential for graduates to have acquired and exited a role over that period. This discrepancy has some implications for the confidence of claims that are possible to be made regarding job roles and earnings; however, the trends present in the years 2012–2017 are replicated in the 2017/2018 data. Finally, it is a limitation that degree qualification outcomes were not gathered, as this would have better illuminated potential differentials between educational outcomes and destinations.

## Conclusion

This study is believed to be the first to offer robust population data regarding the background characteristics and employment outcomes of autistic graduates in United Kingdom as compared to their peers with other conditions or no disabilities. It demonstrates heterogeneity with respect to the university programmes that autistic graduates complete, going beyond the stereotype that all autistic individuals study STEM subjects. It echoes what is known about the wider employment outcomes for autistic adults by providing evidence of persistent unemployment and poorer earnings than peers with Other Disabilities and those with No Disabilities. This evidence ought to be a stimulus for higher education institutions to focus more explicitly on the transitions to employment for autistic students by putting in place provisions and supports to enable their success on completion of their studies.

## Supplemental Material

sj-docx-1-aut-10.1177_13623613231182756 – Supplemental material for Uncovering employment outcomes for autistic university graduates in the United Kingdom: An analysis of population data Supplemental material, sj-docx-1-aut-10.1177_13623613231182756 for Uncovering employment outcomes for autistic university graduates in the United Kingdom: An analysis of population data by Jonathan Vincent and Kevin Ralston in Autism
