# Supplementary material for: Uncovering employment outcomes for autistic university graduates in the United Kingdom: An analysis of population data
Source: Autism. 2023 Jun 23;28(3):732–43. doi: 10.1177/13623613231182756 (PMC10913337; doi:10.1177/13623613231182756)
Supplement: sj-docx-1-aut-10.1177_13623613231182756 – Supplemental material for Uncovering employment outcomes for autistic university graduates in the United Kingdom: An analysis of population data [file sj-docx-1-aut-10.1177_13623613231182756.docx]

**Supplementary Materials**

**Supplementary Table 1 - Subject 1. Degree subject area by self-identified autism status of those working full-time or part-time. Pooled HESA data 2012-2018,** First row has *frequencies* and second row has *column percentages*

|  | **Men** | | | | **Women** | | | |
| --- | --- | --- | --- | --- | --- | --- | --- | --- |
|  | Autism | Other disability | No known disability | Total | Autism | Other disability | No known disability | Total |
| Medicine & dentistry | 10 | 1383 | 10374 | 11767 | 7 | 2032 | 13878 | 15917 |
|  | **0.2%** | **2%** | **2%** | **2%** | **0.6%** | **2%** | **2%** | **2%** |
| Subjects allied to medicine | 77 | 4158 | 26472 | 30707 | 82 | 14103 | 80463 | 94648 |
|  | **2%** | **6%** | **5%** | **5%** | **6%** | **13%** | **13%** | **13%** |
| Biological sciences | 316 | 6358 | 43406 | 50080 | 129 | 12178 | 68653 | 80960 |
|  | **7%** | **9%** | **9%** | **9%** | **10%** | **11%** | **11%** | **11%** |
| Veterinary science | 1 | 101 | 530 | 632 | 2 | 322 | 1330 | 1654 |
|  | **.02%** | **0.1%** | **0.1%** | **0.1%** | **0.2%** | **0.3%** | **0.2%** | **0.2%** |
| Agriculture & related subjects | 38 | 857 | 3844 | 4739 | 27 | 1471 | 7049 | 8547 |
|  | **0.9%** | **1%** | **0.8%** | **0.8%** | **2%** | **1%** | **1%** | **1%** |
| Physical sciences | 336 | 4050 | 27439 | 31825 | 51 | 3590 | 20875 | 24516 |
|  | **8%** | **6%** | **6%** | **6%** | **4%** | **3%** | **3%** | **3%** |
| Mathematical sciences | 204 | 1410 | 13336 | 14950 | 35 | 1163 | 8984 | 10182 |
|  | **5%** | **2%** | **3%** | **3%** | **3%** | **1%** | **1%** | **1%** |
| Computer science | 689 | 4993 | 36505 | 42187 | 45 | 1314 | 8535 | 9894 |
|  | **16%** | **7%** | **7%** | **7%** | **4%** | **1%** | **1%** | **1%** |
| Engineering & technology | 270 | 6361 | 57829 | 64460 | 31 | 1635 | 11415 | 13081 |
|  | **6%** | **9%** | **12%** | **11%** | **2%** | **2%** | **2%** | **2%** |
| Architecture, building & planning | 50 | 2851 | 19991 | 22892 | 5 | 1508 | 10221 | 11734 |
|  | **1%** | **4%** | **4%** | **4%** | **0.4%** | **1%** | **2%** | **2%** |
| Social studies | 294 | 6801 | 45078 | 52173 | 106 | 13028 | 70582 | 83716 |
|  | **7%** | **10%** | **9%** | **9%** | **8%** | **12%** | **11%** | **11%** |
| Law | 79 | 2107 | 16221 | 18407 | 28 | 3485 | 25793 | 29306 |
|  | **2%** | **3%** | **3%** | **3%** | 2% | 3% | 4% | 4% |
| Business & administrative studies | 272 | 7793 | 77300 | 85365 | 57 | 8215 | 77425 | 85697 |
|  | **6%** | **11%** | **15%** | **15%** | **5%** | **8%** | **12%** | **11%** |
| Mass communications & documentation | 256 | 2254 | 14256 | 16766 | 61 | 3068 | 19321 | 22450 |
|  | **6%** | **3%** | **3%** | **3%** | **5%** | **3%** | **3%** | **3%** |
| Languages | 231 | 2429 | 17907 | 20567 | 111 | 6632 | 42928 | 49671 |
|  | **5%** | **3%** | **4%** | **4%** | **9%** | **6%** | **7%** | **7%** |
| Historical & philosophical studies | 323 | 4135 | 23239 | 27697 | 84 | 5393 | 26873 | 32350 |
|  | **7%** | **6%** | **5%** | **5%** | **7%** | **5%** | **4%** | **4%** |
| Creative arts & design | 814 | 9040 | 36749 | 46603 | 288 | 16194 | 59896 | 76378 |
|  | **19%** | **13%** | **7%** | **8%** | **23%** | **15%** | **9%** | **10%** |
| Education | 92 | 3656 | 29588 | 33336 | 112 | 12642 | 79089 | 91843 |
|  | **2%** | **5%** | **6%** | **6%** | **9%** | **12%** | **12%** | **12%** |
| Combined | 14 | 394 | 2699 | 3107 | 7 | 884 | 4280 | 5171 |
|  | **0.3%** | **0.6%** | **0.5%** | **0.5%** | **0.6%** | **0.8%** | **0.7%** | **0.7%** |
| Total | 4366 | 71131 | 502763 | 578260 | 1268 | 108857 | 637590 | 747715 |
|  | 100% | 100% | 100% | 100% | 100% | 100% | 100% | 100% |
|  | | | | |  |  |  |  |

**Supplementary Table 2 - Subject 2. Degree subject area of those who have self-identified with autism by year.**

First row has *frequencies* and second row has *column*

|  | 2012/13 | 2013/14 | 2014/15 | 2015/16 | 2016/17 | 2017/18 | Total |
| --- | --- | --- | --- | --- | --- | --- | --- |
| Medicine & dentistry | 3 | 0 | 2 | 1 | 2 | 9 | 17 |
|  | **0.5%** | **0%** | **0.3%** | **0.1%** | **0.2%** | **0.5%** | **0.3%** |
| Subjects allied to medicine | 16 | 17 | 18 | 22 | 35 | 51 | 159 |
|  | **3%** | **2%** | **2%** | **3%** | **3%** | **3%** | **3%** |
| Biological sciences | 44 | 45 | 52 | 67 | 106 | 131 | 445 |
|  | **7%** | **6%** | **7%** | **8%** | **10%** | **8%** | **8%** |
| Veterinary science | 1 | 0 | 1 | 0 | 0 | 1 | 3 |
|  | **0.2%** | **0%** | **0.1%** | **0%** | **0%** | **.06%** | **.05%** |
| Agriculture & related subjects | 7 | 12 | 11 | 10 | 10 | 15 | 65 |
|  | **1%** | **2%** | **1%** | **1%** | **1%** | **1%** | **1%** |
| Physical sciences | 33 | 52 | 52 | 55 | 83 | 113 | 388 |
|  | **6%** | **7%** | **7%** | **6%** | **8%** | **7%** | **7%** |
| Mathematical sciences | 25 | 32 | 36 | 38 | 46 | 63 | 240 |
|  | **4%** | **5%** | **5%** | **4%** | **4%** | **4%** | **4%** |
| Computer science | 81 | 76 | 92 | 119 | 121 | 246 | 735 |
|  | **14%** | **11%** | **12%** | **14%** | **11%** | **15%** | **13%** |
| Engineering & technology | 31 | 44 | 35 | 51 | 49 | 91 | 301 |
|  | **5%** | **6%** | **5%** | **6%** | **5%** | **5%** | **5%** |
| Architecture, building & planning | 5 | 7 | 7 | 10 | 15 | 11 | 55 |
|  | **1%** | **1%** | **1%** | **1%** | **1%** | **0.6%** | **1%** |
| Social studies | 36 | 53 | 45 | 63 | 87 | 116 | 400 |
|  | **6%** | **8%** | **6%** | **7%** | **8%** | **7%** | **7%** |
| Law | 15 | 14 | 15 | 18 | 18 | 29 | 109 |
|  | **3%** | **2%** | **2%** | **2%** | **2%** | **2%** | **2%** |
| Business & administrative studies | 38 | 38 | 47 | 51 | 72 | 83 | 329 |
|  | **6%** | **5%** | **6%** | **6%** | **7%** | **5%** | **6%** |
| Mass communications & documentation | 35 | 50 | 46 | 39 | 50 | 100 | 320 |
|  | **6%** | **7%** | **6%** | **4%** | **5%** | **6%** | **6%** |
| Languages | 36 | 35 | 63 | 57 | 57 | 94 | 342 |
|  | **6%** | **5%** | **8%** | **7%** | **5%** | **6%** | **6%** |
| Historical & philosophical studies | 42 | 58 | 52 | 69 | 74 | 113 | 408 |
|  | **7%** | **8%** | **7%** | **8%** | **7%** | **7%** | **7%** |
| Creative arts & design | 115 | 134 | 157 | 162 | 199 | 338 | 1105 |
|  | **20%** | **19%** | **21%** | **18%** | **19%** | **20%** | **20%** |
| Education | 18 | 24 | 17 | 43 | 39 | 63 | 204 |
|  | **3%** | **3%** | **2%** | **5%** | **4%** | **4%** | **4%** |
| Combined | 5 | 6 | 3 | 1 | 2 | 4 | 21 |
|  | **0.9%** | **0.9%** | **0.4%** | **0.1%** | **0.2%** | **0.2%** | **0.3%** |
| Total | 586 | 697 | 751 | 876 | 1065 | 1671 | 5646 |
|  | 100% | 100% | 100% | 100% | 100% | 100% | 100% |

**Supplementary Table 3**. Economic activity of graduates 2012 to 2018 by autism for men and women. First row has *frequencies* and second row has *row percentages*

| Self-identified autistic impairment | Men | | | | | |
| --- | --- | --- | --- | --- | --- | --- |
|  | 1. Ft-work | 2. Pt-work | 3. Work+\|study | 4. Unemployed | 5. Other | Total |
| Autism | 1514 | 748 | 1070 | 732 | 302 | 4366 |
|  | 34.68 | 17.13 | 24.51 | 16.77 | 6.92 | 100.00 |
| Other disability | 41374 | 9436 | 11635 | 4893 | 3793 | 71131 |
|  | 58.17 | 13.27 | 16.36 | 6.88 | 5.33 | 100.00 |
| No known disability | 353027 | 59091 | 59410 | 15675 | 15560 | 502763 |
|  | 70.22 | 11.75 | 11.82 | 3.12 | 3.09 | 100.00 |
| Total | 395915 | 69275 | 72115 | 21300 | 19655 | 578260 |
|  | 68.47 | 11.98 | 12.47 | 3.68 | 3.40 | 100.00 |

| Self-identified autistic impairment | Women | | | | | |
| --- | --- | --- | --- | --- | --- | --- |
|  | 1. Ft-work | 2. Pt-work | 3. Work+\|study | 4. Unemployed | 5. Other | Total |
| Autism | 409 | 264 | 346 | 140 | 109 | 1268 |
|  | 32.26 | 20.82 | 27.29 | 11.04 | 8.60 | 100.00 |
| Other disability | 61206 | 18186 | 17578 | 5418 | 6469 | 108857 |
|  | 56.23 | 16.71 | 16.15 | 4.98 | 5.94 | 100.00 |
| No known disability | 417763 | 100804 | 79623 | 16150 | 23250 | 637590 |
|  | 65.52 | 15.81 | 12.49 | 2.53 | 3.65 | 100.00 |
| Total | 479378 | 119254 | 97547 | 21708 | 29828 | 747715 |
|  | 64.11 | 15.95 | 13.05 | 2.90 | 3.99 | 100.00 |
|  | | | | | | |

**Supplementary Table 4**. Economic activity of graduates by autism for each year. First row has *frequencies* and second row has *row percentages*

| Self-identified autistic impairment | **2012/2013** | | | | | |
| --- | --- | --- | --- | --- | --- | --- |
|  | Ft-work | Pt-work | Work+\|study | Unemployed | Other | Total |
| Autism | 165 | 116 | 164 | 118 | 23 | 586 |
|  | 28% | 20% | 28% | 20% | 4% | 100% |
| Other disability | 14368 | 4173 | 4184 | 1734 | 1143 | 25602 |
|  | 56% | 16% | 16% | 7% | 4% | 100% |
| No known disability | 131164 | 29999 | 23539 | 5227 | 4522 | 194451 |
|  | 68% | 15% | 12% | 3% | 3% | 100% |
| Total | 145697 | 34288 | 27887 | 7079 | 5688 | 220639 |
|  | 66% | 16% | 13% | 3% | 3% | 100% |
| Self-identified autistic impairment | **2013/2014** | | | | | |
|  | Ft-work | Pt-work | Work+\|study | Unemployed | Other | Total |
| Autism | 244 | 113 | 174 | 132 | 34 | 697 |
|  | 35% | 16% | 25% | 19% | 5% | 100% |
| Other disability | 15714 | 4190 | 4099 | 1599 | 1261 | 26863 |
|  | 58.50 | 15.60 | 15.26 | 5.95 | 4.69 | 100% |
| No known disability | 131098 | 27672 | 21542 | 4807 | 4551 | 189670 |
|  | 69% | 15% | 11% | 3% | 2% | 100% |
| Total | 147056 | 31975 | 25815 | 6538 | 5846 | 217230 |
|  | 68% | 15% | 12% | 3% | 3% | 100% |
| Self-identified autistic impairment | **2014/2015** | | | | | |
|  | Ft-work | Pt-work | Work+\|study | Unemployed | Other | Total |
| Autism | 239 | 167 | 183 | 127 | 35 | 751 |
|  | 31.82 | 22.24 | 24.37 | 16.91 | 4.66 | 100.00 |
| Other disability | 15763 | 4601 | 4106 | 1521 | 1184 | 27175 |
|  | 58.01 | 16.93 | 15.11 | 5.60 | 4.36 | 100.00 |
| No known disability | 124459 | 26480 | 18976 | 4584 | 4178 | 178677 |
|  | 69.66 | 14.82 | 10.62 | 2.57 | 2.34 | 100.00 |
| Total | 140461 | 31248 | 23265 | 6232 | 5397 | 206603 |
|  | 67.99 | 15.12 | 11.26 | 3.02 | 2.61 | 100.00 |

| Self-identified autistic impairment | **2015/2016** | | | | | |
| --- | --- | --- | --- | --- | --- | --- |
|  | Ft-work | Pt-work | Work+\|study | Unemployed | Other | Total |
| Autism | 283 | 164 | 231 | 150 | 48 | 876 |
|  | 32% | 19% | 26% | 17% | 5% | 100% |
| Other disability | 15920 | 4441 | 4285 | 1527 | 1233 | 27406 |
|  | 58% | 16% | 16% | 6% | 5% | 100% |
| No known disability | 119939 | 25309 | 18583 | 4400 | 4111 | 172342 |
|  | 70% | 15 | 11% | 3% | 2% | 100% |
| Total | 136142 | 29914 | 23099 | 6077 | 5392 | 200624 |
|  | 68% | 15% | 12% | 3% | 3% | 100% |

**Supplementary Table 4** (continued). Economic activity of graduates by autism for each year. First row has *frequencies* and second row has *row percentages*

| Self-identified autistic impairment | **2016/2017** | | | | | | | |
| --- | --- | --- | --- | --- | --- | --- | --- | --- |
|  | Ft-work | Pt-work | | | Work+\|study | Unemployed | Other | Total |
| Autism | 386 | | 183 | 295 | | 140 | 61 | 1065 |
|  | 36% | | 17% | 28% | | 13% | 6% | 100% |
| Other disability | 17303 | | 4745 | 4653 | | 1547 | 1303 | 29551 |
|  | 59% | | 16% | 16% | | 5% | 4% | 100% |
| No known disability | 120863 | | 25097 | 18618 | | 4403 | 3882 | 172863 |
|  | 70% | | 15% | 11% | | 3% | 2% | 100% |
| Total | 138552 | | 30025 | 23566 | | 6090 | 5246 | 203479 |
|  | 68% | | 15% | 12% | | 3% | 3% | 100% |
| Self-identified autistic impairment | **2017/2018** | | | | | | | |
|  | Ft-work | | Pt-work | | Work+\|study | Unemployed | Other | Total |
| Autism | 608 | | 269 | 375 | | 207 | 212 | 1671 |
|  | 36% | | 16% | 22% | | 12% | 13% | 100% |
| Other disability | 23561 | | 5496 | 7935 | | 2393 | 4151 | 43536 |
|  | 54% | | 13% | 18% | | 6% | 10% | 100% |
| No known disability | 143391 | | 25369 | 37864 | | 8430 | 17580 | 232634 |
|  | 62% | | 11% | 16% | | 4% | 8% | 100% |
| Total | 167560 | | 31134 | 46174 | | 11030 | 21943 | 277841 |
|  | 60% | | 11% | 17% | | 4% | 8% | 100% |
|  | | | | | | | | |

**Supplementary Table 5.** Income by whether and individual self-identified with autism for men. First row has *frequencies* and second row has *row percentages*

| Self-identified autistic impairment | **2012/2013 income, men** | | | |
| --- | --- | --- | --- | --- |
|  | <£20,000 | £20,001 - £25,000 | >£25,001 | Total |
| Autism | 36 | 19 | 23 | 78 |
|  | 46% | 24% | 29% | 100% |
| Other disability | 1665 | 1042 | 1152 | 3859 |
|  | 43% | 27% | 30% | 100% |
| No known disability | 15825 | 9576 | 14302 | 39703 |
|  | 40% | 24% | 36% | 100% |
| Total | 17526 | 10637 | 15477 | 43640 |
|  | 40% | 24% | 35% | 100% |
|  | **2014/2015 income, men** | | | |
|  | <£20,000 | £20,001 -£25,000 | >£25,001 | Total |
| Autism | 71 | 28 | 31 | 130 |
|  | 55% | 22% | 24% | 100% |
| Other disability | 1707 | 1295 | 1457 | 4459 |
|  | 38% | 29% | 33% | 100% |
| No known disability | 13347 | 9960 | 15414 | 38721 |
|  | 34% | 26% | 40% | 100% |
| Total | 15125 | 11283 | 16902 | 43310 |
|  | 35% | 26% | 39% | 100% |
|  | **2015/2016 income, men** | | | |
|  | <£20,000 | £20,001 - £25,000 | >£25,001 | Total |
| Autism | 66 | 46 | 31 | 143 |
|  | 46.15 | 32.17 | 21.68 | 100.00 |
| Other disability | 1534 | 1269 | 1527 | 4330 |
|  | 35.43 | 29.31 | 35.27 | 100.00 |
| No known disability | 12346 | 9627 | 15093 | 37066 |
|  | 33.31 | 25.97 | 40.72 | 100.00 |
| Total | 13946 | 10942 | 16651 | 41539 |
|  | 33.57 | 26.34 | 40.09 | 100.00 |
|  | **2016/2017 income, men** | | | |
|  | <£20,000 | £20,001 - £25,000 | >£25,001 | Total |
| Autism | 102 | 43 | 41 | 186 |
|  | 54.84 | 23.12 | 22.04 | 100.00 |
| Other disability | 1654 | 1240 | 1664 | 4558 |
|  | 36.29 | 27.20 | 36.51 | 100.00 |
| No known disability | 11191 | 9827 | 15884 | 36902 |
|  | 30.33 | 26.63 | 43.04 | 100.00 |
| Total | 12947 | 11110 | 17589 | 41646 |
|  | 31.09 | 26.68 | 42.23 | 100.00 |
|  | **2017/2018 income, men** | | | |
|  | <£20,000 | £20,001 - £25,000 | >£25,001 | Total |
| Autism | 144 | 102 | 126 | 372 |
|  | 38.71 | 27.42 | 33.87 | 100.00 |
| Other disability | 1874 | 2137 | 3522 | 7533 |
|  | 24.88 | 28.37 | 46.75 | 100.00 |
| No known disability | 11708 | 13716 | 29419 | 54843 |
|  | 21.35 | 25.01 | 53.64 | 100.00 |
| Total | 13726 | 15955 | 33067 | 62748 |
|  | 21.87 | 25.43 | 52.70 | 100.00 |

**Supplementary Table 6.** Income by whether and individual self-identified with autism for women. First row has *frequencies* and second row has *row percentages*

| Self-identified autistic impairment | **2012/2013 income, women** | | | |
| --- | --- | --- | --- | --- |
|  | <£20,000 | £20,001 - £25,000 | >£25,001 | Total |
| Autism | 15 | 8 | 8 | 31 |
|  | 48% | 26% | 26% | 100% |
| Other disability | 2783 | 1749 | 1114 | 5646 |
|  | 49% | 31% | 20% | 100% |
| No known disability | 24221 | 13051 | 12507 | 49779 |
|  | 49% | 26% | 25% | 100% |
| Total | 27019 | 14808 | 13629 | 55456 |
|  | 48% | 27% | 25% | 100% |
|  | **2014/2015 income, women** | | | |
|  | <£20,000 | £20,001 -£25,000 | >£25,001 | Total |
| Autism | 13 | 7 | 9 | 29 |
|  | 45% | 24% | 31% | 100% |
| Other disability | 2922 | 2084 | 1531 | 6537 |
|  | 45% | 32% | 23% | 100% |
| No known disability | 20846 | 13322 | 13555 | 47723 |
|  | 44% | 28% | 28% | 100% |
| Total | 23781 | 15413 | 15095 | 54289 |
|  | 44% | 28% | 28% | 100% |
|  | **2015/2016 income, women** | | | |
|  | <£20,000 | £20,001 - £25,000 | >£25,001 | Total |
| Autism | 24 | 10 | 9 | 43 |
|  | 56% | 23% | 21% | 100% |
| Other disability | 2972 | 2207 | 1652 | 6831 |
|  | 44% | 32% | 24% | 100% |
| No known disability | 19591 | 12848 | 13300 | 45739 |
|  | 43% | 28% | 29% | 100% |
| Total | 22587 | 15065 | 14961 | 52613 |
|  | 43% | 29% | 28% | 100% |
|  | **2016/2017 income, women** | | | |
|  | <£20,000 | £20,001 - £25,000 | >£25,001 | Total |
| Autism | 26 | 16 | 21 | 63 |
|  | 41% | 25% | 33% | 100% |
| Other disability | 3063 | 2452 | 2006 | 7521 |
|  | 41% | 33% | 27% | 100% |
| No known disability | 18413 | 13309 | 14347 | 46069 |
|  | 40% | 29% | 31% | 100% |
| Total | 21502 | 15777 | 16374 | 53653 |
|  | 40% | 29% | 31% | 100% |
|  | \| **2017/2018 income, women** \| \| --- \| | | | |
|  | <£20,000 | £20,001 - £25,000 | >£25,001 | Total |
| Autism | 26 | 16 | 21 | 63 |
|  | 41% | 25% | 33% | 100% |
| Other disability | 3063 | 2452 | 2006 | 7521 |
|  | 41% | 33% | 27% | 100% |
| No known disability | 18413 | 13309 | 14347 | 46069 |
|  | 40% | 29% | 31% | 100% |
| Total | 21502 | 15777 | 16374 | 53653 |
|  | 40% | 29% | 31% | 100% |
|  | | | | |

**Table 7 SIC. Men, standard industrial classification by self-identified autism status of those working full-time or part-time. Pooled HESA data 2012-2018,** First row has *frequencies* and second row has *column percentages*

| Standard Industrial Classification | An impairment such as an autistic spectrum disorder | | | |
| --- | --- | --- | --- | --- |
|  | Autism | Other disability | No known disability | Total |
| AGRICULTURE, FORESTRY AND FISHING | 5 | 268 | 1526 | 1799 |
|  | **0.2%** | **0.5%** | **0.4%** | **0.4%** |
| MINING AND QUARRYING | 8 | 251 | 3352 | 3611 |
|  | **0.4%** | **0.5%** | **0.8%** | **0.8%** |
| MANUFACTURING | 118 | 3125 | 31621 | 34864 |
|  | **5%** | **6%** | **8%** | **8%** |
| ELECTRICITY, GAS etc | 10 | 255 | 2979 | 3244 |
|  | **0.4%** | **0.5%** | **0.7%** | **0.7%** |
| WATER SUPPLY; SEWERAGE etc | 4 | 156 | 1832 | 1992 |
|  | **0.2%** | **0.3%** | **0.4%** | **0.4%** |
| CONSTRUCTION | 40 | 1494 | 13922 | 15456 |
|  | **2%** | **3%** | **3%** | **3%** |
| WHOLESALE AND RETAIL vehicles | 322 | 4961 | 40466 | 45749 |
|  | **14%** | **10%** | **10%** | **10%** |
| TRANSPORTATION AND STORAGE | 33 | 854 | 8598 | 9485 |
|  | **1%** | **2%** | **2%** | **2%** |
| ACCOMMODATION AND FOOD SERVICE | 139 | 2522 | 18499 | 21160 |
|  | **6%** | **5%** | **4%** | **5%** |
| INFORMATION AND COMMUNICATION | 333 | 5148 | 40421 | 45902 |
|  | **15%** | **10%** | **10%** | **10%** |
| FINANCIAL AND INSURANCE | 71 | 2173 | 24283 | 26527 |
|  | **3%** | **4%** | **6%** | **6%** |
| REAL ESTATE ACTIVITIES | 16 | 679 | 4893 | 5588 |
|  | **0.7%** | **1%** | **1%** | **1%** |
| PROFESSIONAL, SCIENTIFIC AND TECHNICAL | 205 | 6652 | 56986 | 63843 |
|  | **9%** | **13%** | **14%** | **14%** |
| ADMINISTRATIVE AND SUPPORT SERVICE | 106 | 2282 | 18428 | 20816 |
|  | **4%** | **4%** | **4%** | **4%** |
| PUBLIC ADMINISTRATION AND DEFENCE | 96 | 2541 | 21935 | 24572 |
|  | **4%** | **5%** | **5%** | **5%** |
| EDUCATION | 347 | 7858 | 60893 | 69098 |
|  | **15%** | **15%** | **15%** | **15%** |
| HUMAN HEALTH AND SOCIAL WORK | 163 | 5588 | 37404 | 43155 |
|  | **7%** | **11%** | **9%** | **9%** |
| ARTS, ENTERTAINMENT | 202 | 3162 | 18585 | 21949 |
|  | **9%** | **6%** | **5%** | **5%** |
| OTHER SERVICE | 40 | 763 | 4800 | 5603 |
|  | **2%** | **2%** | **1%** | **1%** |
| HOUSEHOLDS AS EMPLOYERS | 1 | 23 | 122 | 146 |
|  | **0.04%** | **0.05%** | **0.03%** | **0.03%** |
| extraterritorial organisations | 3 | 55 | 573 | 631 |
|  | **0.1%** | **0.1%** | **0.1%** | **0.1%** |
| Total | 2262 | 50810 | 412118 | 465190 |
|  | 100% | 100% | 100% | 100% |
| Source: Destinations of Leavers from Higher Education survey (DLHE) 2012 to 2017  and the Graduate Outcomes Survey (GOS) in 2018 | | | | |

**Table 8 SIC. Women, standard industrial classification by self-identified autism status of those working full-time or part-time. Pooled HESA data 2012-2018,** First row has *frequencies* and second row has *column*

| Standard Industrial Classification | An impairment such as an autistic spectrum disorder | | | |
| --- | --- | --- | --- | --- |
|  | Autism | Other disability | No known disability | Total |
| AGRICULTURE, FORESTRY AND FISHING | 1 | 147 | 890 | 1038 |
|  | **0.2%** | **0.2%** | **0.2%** | **0.2%** |
| MINING AND QUARRYING | 0 | 100 | 1426 | 1526 |
|  | **0** | **0.1%** | **0.3%** | **0.3%** |
| MANUFACTURING | 16 | 2499 | 18674 | 21189 |
|  | **2%** | **3%** | **4%** | **4%** |
| ELECTRICITY, GAS etc | 2 | 190 | 1651 | 1843 |
|  | **0.3%** | **0.2%** | **0.3%** | **0.3%** |
| WATER SUPPLY; SEWERAGE etc | 3 | 172 | 1247 | 1422 |
|  | **0.5%** | **0.2%** | **0.2%** | **0.2%** |
| CONSTRUCTION | 3 | 670 | 4987 | 5660 |
|  | **0.5%** | **0.8%** | **1%** | **1%** |
| WHOLESALE AND RETAIL vehicles | 103 | 8220 | 54218 | 62541 |
|  | **15%** | **10%** | **11%** | **11%** |
| TRANSPORTATION AND STORAGE | 6 | 592 | 5187 | 5785 |
|  | **1%** | **1%** | **1%** | **1%** |
| ACCOMMODATION AND FOOD SERVICE | 25 | 3658 | 23499 | 27182 |
|  | **4%** | **5%** | **5%** | **5%** |
| INFORMATION AND COMMUNICATION | 48 | 3509 | 25282 | 28839 |
|  | **7%** | **4%** | **5%** | **5%** |
| FINANCIAL AND INSURANCE | 20 | 2056 | 18646 | 20722 |
|  | **3%** | **3%** | **4%** | **4%** |
| REAL ESTATE ACTIVITIES | 3 | 908 | 6223 | 7134 |
|  | **0.5%** | **1%** | **1%** | **1%** |
| PROFESSIONAL, SCIENTIFIC AND TECHNICAL | 31 | 7497 | 56209 | 63737 |
|  | **5%** | **9%** | **11%** | **11%** |
| ADMINISTRATIVE AND SUPPORT SERVICE | 22 | 3105 | 21301 | 24428 |
|  | **3%** | **4%** | **4%** | **4%** |
| PUBLIC ADMINISTRATION AND DEFENCE | 38 | 4877 | 29880 | 34795 |
|  | **6%** | **6%** | **6%** | **6%** |
| EDUCATION | 175 | 17368 | 111227 | 128770 |
|  | **26%** | **23%** | **22%** | **22%** |
| HUMAN HEALTH AND SOCIAL WORK | 98 | 18169 | 108386 | 126653 |
|  | **15%** | **23%** | **21%** | **21%** |
| ARTS, ENTERTAINMENT | 62 | 4039 | 19905 | 24006 |
|  | **9%** | **5%** | **4%** | **4%** |
| OTHER SERVICE | 15 | 1386 | 7974 | 9375 |
|  | **2%** | **2%** | **2%** | **2%** |
| HOUSEHOLDS AS EMPLOYERS | 2 | 179 | 891 | 1072 |
|  | **0.3%** | **0.2%** | **0.2%** | **0.2%** |
| extraterritorial organisations | 0 | 51 | 864 | 915 |
|  | **0** | **0.1%** | **0.2%** | **0.2%** |
| Total | 673 | 79392 | 518567 | 598632 |
|  | 100% | 100% | 100% | 100% |
| Source: Destinations of Leavers from Higher Education survey (DLHE) 2012 to 2017  and the Graduate Outcomes Survey (GOS) in 2018 | | | | |

**Supplementary Table 9 - SIC 1. Standard industrial classification by self-identified autism status of those working full-time or part-time. Pooled HESA data 2012-2018,** First row has *frequencies* and second row has *column percentages*

| One Digit Standard Industrial Classification |  | | | |
| --- | --- | --- | --- | --- |
|  | Autism | Other disability | No known disability | Total |
| AGRICULTURE, FORESTRY AND FISHING | 6 | 415 | 2416 | 2837 |
|  | **0.2%** | **0.3%** | **0.3%** | **0.3%** |
| MINING AND QUARRYING | 8 | 351 | 4778 | 5137 |
|  | **0.3%** | **0.3%** | **0.5%** | **0.5%** |
| MANUFACTURING | 134 | 5629 | 50296 | 56059 |
|  | **4.6%** | **4.3%** | **5.4%** | **5.3%** |
| ELECTRICITY, GAS etc | 12 | 445 | 4631 | 5088 |
|  | **0.4%** | **0.3%** | **0.5%** | **0.5%** |
| WATER SUPPLY; SEWERAGE etc | 7 | 329 | 3079 | 3415 |
|  | **0.2%** | **0.3%** | **0.3%** | **0.3%** |
| CONSTRUCTION | 43 | 2164 | 18910 | 21117 |
|  | **1.5%** | **1.7%** | **2.0%** | **2%** |
| WHOLESALE AND RETAIL vehicles servicing | 425 | 13190 | 94698 | 108313 |
|  | **14.5%** | **10.1%** | **10.2%** | **10.2%** |
| TRANSPORTATION AND STORAGE | 39 | 1447 | 13787 | 15273 |
|  | **1.3%** | **1.1%** | **1.5%** | **1.4%** |
| ACCOMMODATION AND FOOD SERVICE | 164 | 6184 | 42004 | 48352 |
|  | **5.6%** | **4.8%** | **4.5%** | **4.5%** |
| INFORMATION AND COMMUNICATION | 381 | 8668 | 65717 | 74766 |
|  | **13%** | **6.7%** | **7%** | **7%** |
| FINANCIAL AND INSURANCE | 91 | 4231 | 42936 | 47258 |
|  | **3.1%** | **3.3%** | **4.6%** | **4.4%** |
| REAL ESTATE ACTIVITIES | 19 | 1587 | 11119 | 12725 |
|  | **0.7%** | **1.2%** | **1.2%** | **1.2%** |
| PROFESSIONAL, SCIENTIFIC AND TECHNICAL | 236 | 14155 | 113222 | 127613 |
|  | **8%** | **10.9%** | **12%** | **12%** |
| ADMINISTRATIVE AND SUPPORT SERVICE | 129 | 5389 | 39733 | 45251 |
|  | **4.4%** | **4.1%** | **4.3%** | **4.3%** |
| PUBLIC ADMINISTRATION AND DEFENCE | 134 | 7419 | 51822 | 59375 |
|  | **4.6%** | **5.7%** | **5.6%** | **5.6%** |
| EDUCATION | 523 | 25242 | 172157 | 197922 |
|  | **17.8%** | **19.4%** | **18.5%** | **18.6%** |
| HUMAN HEALTH AND SOCIAL WORK | 261 | 23760 | 145806 | 169827 |
|  | **9%** | **18.2%** | **16%** | **16%** |
| ARTS, ENTERTAINMENT | 264 | 7210 | 38501 | 45975 |
|  | **9%** | **5.5%** | **4.1%** | **4.3%** |
| OTHER SERVICE | 55 | 2152 | 12778 | 14985 |
|  | **1.9%** | **1.7%** | **1.4%** | **1.4%** |
| HOUSEHOLDS AS EMPLOYERS | 3 | 202 | 1013 | 1218 |
|  | **0.1%** | **0.2%** | **0.1%** | **0.1%** |
| extraterritorial organisations | 3 | 106 | 1437 | 1546 |
|  | **0.1%** | **0.1%** | **0.2%** | **0.2%** |
| Total | 2937 | 130275 | 930840 | 1064052 |
|  | 100% | 100% | 100% | 100% |
|  | | | | |

**Supplementary Table 10 - SIC 2. Men, standard industrial classification by self-identified autism status of those working full-time or part-time. Pooled HESA data 2012-2018,** First row has *frequencies* and second row has *column percentages*

| Standard Industrial Classification | An impairment such as an autistic spectrum disorder | | | |
| --- | --- | --- | --- | --- |
|  | Autism | Other disability | No known disability | Total |
| AGRICULTURE, FORESTRY AND FISHING | 5 | 268 | 1526 | 1799 |
|  | **0.2%** | **0.5%** | **0.4%** | **0.4%** |
| MINING AND QUARRYING | 8 | 251 | 3352 | 3611 |
|  | **0.4%** | **0.5%** | **0.8%** | **0.8%** |
| MANUFACTURING | 118 | 3125 | 31621 | 34864 |
|  | **5%** | **6%** | **8%** | **8%** |
| ELECTRICITY, GAS etc | 10 | 255 | 2979 | 3244 |
|  | **0.4%** | **0.5%** | **0.7%** | **0.7%** |
| WATER SUPPLY; SEWERAGE etc | 4 | 156 | 1832 | 1992 |
|  | **0.2%** | **0.3%** | **0.4%** | **0.4%** |
| CONSTRUCTION | 40 | 1494 | 13922 | 15456 |
|  | **2%** | **3%** | **3%** | **3%** |
| WHOLESALE AND RETAIL vehicles | 322 | 4961 | 40466 | 45749 |
|  | **14%** | **10%** | **10%** | **10%** |
| TRANSPORTATION AND STORAGE | 33 | 854 | 8598 | 9485 |
|  | **1%** | **2%** | **2%** | **2%** |
| ACCOMMODATION AND FOOD SERVICE | 139 | 2522 | 18499 | 21160 |
|  | **6%** | **5%** | **4%** | **5%** |
| INFORMATION AND COMMUNICATION | 333 | 5148 | 40421 | 45902 |
|  | **15%** | **10%** | **10%** | **10%** |
| FINANCIAL AND INSURANCE | 71 | 2173 | 24283 | 26527 |
|  | **3%** | **4%** | **6%** | **6%** |
| REAL ESTATE ACTIVITIES | 16 | 679 | 4893 | 5588 |
|  | **0.7%** | **1%** | **1%** | **1%** |
| PROFESSIONAL, SCIENTIFIC AND TECHNICAL | 205 | 6652 | 56986 | 63843 |
|  | **9%** | **13%** | **14%** | **14%** |
| ADMINISTRATIVE AND SUPPORT SERVICE | 106 | 2282 | 18428 | 20816 |
|  | **4%** | **4%** | **4%** | **4%** |
| PUBLIC ADMINISTRATION AND DEFENCE | 96 | 2541 | 21935 | 24572 |
|  | **4%** | **5%** | **5%** | **5%** |
| EDUCATION | 347 | 7858 | 60893 | 69098 |
|  | **15%** | **15%** | **15%** | **15%** |
| HUMAN HEALTH AND SOCIAL WORK | 163 | 5588 | 37404 | 43155 |
|  | **7%** | **11%** | **9%** | **9%** |
| ARTS, ENTERTAINMENT | 202 | 3162 | 18585 | 21949 |
|  | **9%** | **6%** | **5%** | **5%** |
| OTHER SERVICE | 40 | 763 | 4800 | 5603 |
|  | **2%** | **2%** | **1%** | **1%** |
| HOUSEHOLDS AS EMPLOYERS | 1 | 23 | 122 | 146 |
|  | **0.04%** | **0.05%** | **0.03%** | **0.03%** |
| extraterritorial organisations | 3 | 55 | 573 | 631 |
|  | **0.1%** | **0.1%** | **0.1%** | **0.1%** |
| Total | 2262 | 50810 | 412118 | 465190 |
|  | 100% | 100% | 100% | 100% |
|  | | | | |
|  | | | | |

**Supplementary Table 11 - SIC 3. Women, standard industrial classification by self-identified autism status of those working full-time or part-time. Pooled HESA data 2012-2018,** First row has *frequencies* and second row has *column*

| Standard Industrial Classification | An impairment such as an autistic spectrum disorder | | | |
| --- | --- | --- | --- | --- |
|  | Autism | Other disability | No known disability | Total |
| AGRICULTURE, FORESTRY AND FISHING | 1 | 147 | 890 | 1038 |
|  | **0.2%** | **0.2%** | **0.2%** | **0.2%** |
| MINING AND QUARRYING | 0 | 100 | 1426 | 1526 |
|  | **0** | **0.1%** | **0.3%** | **0.3%** |
| MANUFACTURING | 16 | 2499 | 18674 | 21189 |
|  | **2%** | **3%** | **4%** | **4%** |
| ELECTRICITY, GAS etc | 2 | 190 | 1651 | 1843 |
|  | **0.3%** | **0.2%** | **0.3%** | **0.3%** |
| WATER SUPPLY; SEWERAGE etc | 3 | 172 | 1247 | 1422 |
|  | **0.5%** | **0.2%** | **0.2%** | **0.2%** |
| CONSTRUCTION | 3 | 670 | 4987 | 5660 |
|  | **0.5%** | **0.8%** | **1%** | **1%** |
| WHOLESALE AND RETAIL vehicles | 103 | 8220 | 54218 | 62541 |
|  | **15%** | **10%** | **11%** | **11%** |
| TRANSPORTATION AND STORAGE | 6 | 592 | 5187 | 5785 |
|  | **1%** | **1%** | **1%** | **1%** |
| ACCOMMODATION AND FOOD SERVICE | 25 | 3658 | 23499 | 27182 |
|  | **4%** | **5%** | **5%** | **5%** |
| INFORMATION AND COMMUNICATION | 48 | 3509 | 25282 | 28839 |
|  | **7%** | **4%** | **5%** | **5%** |
| FINANCIAL AND INSURANCE | 20 | 2056 | 18646 | 20722 |
|  | **3%** | **3%** | **4%** | **4%** |
| REAL ESTATE ACTIVITIES | 3 | 908 | 6223 | 7134 |
|  | **0.5%** | **1%** | **1%** | **1%** |
| PROFESSIONAL, SCIENTIFIC AND TECHNICAL | 31 | 7497 | 56209 | 63737 |
|  | **5%** | **9%** | **11%** | **11%** |
| ADMINISTRATIVE AND SUPPORT SERVICE | 22 | 3105 | 21301 | 24428 |
|  | **3%** | **4%** | **4%** | **4%** |
| PUBLIC ADMINISTRATION AND DEFENCE | 38 | 4877 | 29880 | 34795 |
|  | **6%** | **6%** | **6%** | **6%** |
| EDUCATION | 175 | 17368 | 111227 | 128770 |
|  | **26%** | **23%** | **22%** | **22%** |
| HUMAN HEALTH AND SOCIAL WORK | 98 | 18169 | 108386 | 126653 |
|  | **15%** | **23%** | **21%** | **21%** |
| ARTS, ENTERTAINMENT | 62 | 4039 | 19905 | 24006 |
|  | **9%** | **5%** | **4%** | **4%** |
| OTHER SERVICE | 15 | 1386 | 7974 | 9375 |
|  | **2%** | **2%** | **2%** | **2%** |
| HOUSEHOLDS AS EMPLOYERS | 2 | 179 | 891 | 1072 |
|  | **0.3%** | **0.2%** | **0.2%** | **0.2%** |
| extraterritorial organisations | 0 | 51 | 864 | 915 |
|  | **0** | **0.1%** | **0.2%** | **0.2%** |
| Total | 673 | 79392 | 518567 | 598632 |
|  | 100% | 100% | 100% | 100% |

**Supplementary Table 12 - SIC Standard industrial classification by self-identified autism status of those working full-time or part-time by year 2012-2018,** First row has *frequencies* and second row has *column*

| Standard Industrial Classification | Year | | | | | | |
| --- | --- | --- | --- | --- | --- | --- | --- |
|  | 2012/13 | 2013/14 | 2014/15 | 2015/16 | 2016/17 | 2017/18 | Total |
| AGRICULTURE, FORESTRY AND FISHING | 0 | 0 | 1 | 1 | 3 | 1 | 6 |
|  | 0.00 | 0.00 | 0.25% | 0.22% | 0.53% | 0.11% | 0.20% |
| MINING AND QUARRYING | 0 | 0 | 2 | 1 | 3 | 2 | 8 |
|  | 0.00 | 0.00 | 0.49% | 0.22% | 0.53% | 0.23% | 0.27% |
| MANUFACTURING | 9 | 18 | 8 | 19 | 37 | 43 | 134 |
|  | 3.20% | 5.04% | 1.97% | 4.25% | 6.50% | 4.90% | 4.56% |
| ELECTRICITY, GAS etc | 1 | 0 | 2 | 4 | 0 | 5 | 12 |
|  | 0.36% | 0.00 | 0.49% | 0.89% | 0.00 | 0.57% | 0.41% |
| WATER SUPPLY; SEWERAGE etc | 2 | 1 | 1 | 0 | 3 | 0 | 7 |
|  | 0.71% | 0.28% | 0.25% | 0.00 | 0.53% | 0.00 | 0.24% |
| CONSTRUCTION | 3 | 9 | 6 | 4 | 9 | 12 | 43 |
|  | 1.07% | 2.52% | 1.48% | 0.89% | 1.58% | 1.37% | 1.46% |
| WHOLESALE AND RETAIL vehicles | 47 | 56 | 65 | 81 | 81 | 95 | 425 |
|  | 16.73% | 15.69% | 16.01% | 18.12% | 14.24% | 10.83% | 14.47% |
| TRANSPORTATION AND STORAGE | 4 | 4 | 4 | 8 | 5 | 14 | 39 |
|  | 1.42% | 1.12% | 0.99% | 1.79% | 0.88% | 1.60% | 1.33% |
| ACCOMMODATION AND FOOD SERVICE | 8 | 27 | 26 | 32 | 35 | 36 | 164 |
|  | 2.85% | 7.56% | 6.40% | 7.16% | 6.15% | 4.10% | 5.58% |
| INFORMATION AND COMMUNICATION | 30 | 52 | 53 | 59 | 68 | 119 | 381 |
|  | 10.68% | 14.57% | 13.05% | 13.20% | 11.95% | 13.57% | 12.97% |
| FINANCIAL AND INSURANCE | 10 | 8 | 11 | 13 | 17 | 32 | 91 |
|  | 3.56% | 2.24% | 2.71% | 2.91% | 2.99% | 3.65% | 3.10% |
| REAL ESTATE ACTIVITIES | 0 | 2 | 1 | 2 | 7 | 7 | 19 |
|  | 0.00 | 0.56% | 0.25% | 0.45% | 1.23% | 0.80% | 0.65% |
| PROFESSIONAL, SCIENTIFIC AND TECHNICAL | 24 | 23 | 41 | 28 | 40 | 80 | 236 |
|  | 8.54% | 6.44% | 10.10% | 6.26% | 7.03% | 9.12% | 8.04% |
| ADMINISTRATIVE AND SUPPORT SERVICE | 16 | 23 | 16 | 20 | 20 | 34 | 129 |
|  | 5.69% | 6.44% | 3.94% | 4.47% | 3.51% | 3.88% | 4.39% |
| PUBLIC ADMINISTRATION AND DEFENCE | 15 | 14 | 20 | 22 | 27 | 36 | 134 |
|  | 5.34% | 3.92% | 4.93% | 4.92% | 4.75% | 4.10% | 4.56% |
| EDUCATION | 49 | 41 | 50 | 78 | 96 | 209 | 523 |
|  | 17.44% | 11.48% | 12.32% | 17.45% | 16.87% | 23.83% | 17.81% |
| HUMAN HEALTH AND SOCIAL WORK | 28 | 40 | 48 | 26 | 59 | 60 | 261 |
|  | 9.96% | 11.20% | 11.82% | 5.82% | 10.37% | 6.84% | 8.89% |
| ARTS, ENTERTAINMENT | 33 | 22 | 44 | 39 | 50 | 76 | 264 |
|  | 11.74% | 6.16% | 10.84% | 8.72% | 8.79% | 8.67% | 8.99% |
| OTHER SERVICE | 2 | 13 | 7 | 10 | 8 | 15 | 55 |
|  | 0.71% | 3.64% | 1.72% | 2.24% | 1.41% | 1.71% | 1.87% |
| HOUSEHOLDS AS EMPLOYERS | 0 | 2 | 0 | 0 | 1 | 0 | 3 |
|  | 0.00 | 0.56% | 0.00 | 0.00 | 0.18 | 0.00 | 0.10% |
| extraterritorial organisations | 0 | 2 | 0 | 0 | 0 | 1 | 3 |
|  | 0.00 | 0.56% | 0.00 | 0.00 | 0.00 | 0.11% | 0.10% |
| Total | 281 | 357 | 406 | 447 | 569 | 877 | 2937 |
|  | 100% | 100% | 100% | 100% | 100% | 100% | 100% |
|  | | | | | | | |
